# Lck is a relevant target in chronic lymphocytic leukaemia cells whose expression variance is unrelated to disease outcome

**DOI:** 10.1038/s41598-017-17021-w

**Published:** 2017-12-01

**Authors:** Kathleen J. Till, John C. Allen, Fatima Talab, Ke Lin, David Allsup, Lynn Cawkwell, Alison Bentley, Ingo Ringshausen, Andrew D. Duckworth, Andrew R. Pettitt, Nagesh Kalakonda, Joseph R. Slupsky

**Affiliations:** 1 Department of Molecular and Clinical Cancer Medicine, University of Liverpool, Liverpool, UK; 2 Department of Haematology, Royal Liverpool and Broadgreen University Hospitals NHS Trust, Liverpool, UK; 3 Department of Haematology, Queens Centre for Oncology and Haematology, Hull and East Yorkshire Hospitals NHS Trust, Yorkshire, UK; 4 School of Life Sciences, University of Hull, Hull, UK; 5 Hull York Medical School, University of Hull, Hull, UK; 6 Department of Haematology, University of Cambridge, Cambridge, UK

## Abstract

Pathogenesis of chronic lymphocytic leukaemia (CLL) is contingent upon antigen receptor (BCR) expressed by malignant cells of this disease. Studies on somatic hypermutation of the antigen binding region, receptor expression levels and signal capacity have all linked BCR on CLL cells to disease prognosis. Our previous work showed that the src-family kinase Lck is a targetable mediator of BCR signalling in CLL cells, and that variance in Lck expression associated with ability of BCR to induce signal upon engagement. This latter finding makes Lck similar to ZAP70, another T-cell kinase whose aberrant expression in CLL cells also associates with BCR signalling capacity, but also different because ZAP70 is not easily pharmacologically targetable. Here we describe a robust method of measuring Lck expression in CLL cells using flow cytometry. However, unlike ZAP70 whose expression in CLL cells predicts prognosis, we find Lck expression and disease outcome in CLL are unrelated despite observations that its inhibition produces effects that biologically resemble the egress phenotype taken on by CLL cells treated with idelalisib. Taken together, our findings provide insight into the pathobiology of CLL to suggest a more complex relationship between expression of molecules within the BCR signalling pathway and disease outcome.

## Introduction

Chronic lymphocytic leukaemia (CLL) is a heterogeneous malignancy of mature B lymphocytes. This disease is important because it is a common leukaemia among elderly adults in North America and Europe, and because of the significant morbidity and mortality associated with the progressive form of this disease. Several biomarkers have been identified that distinguish between indolent and progressive disease in CLL, and each has advantages and disadvantages according to the clinical information provided and ease of measurement^1^. However, none of these markers are useful for patient stratification with respect to the recent introduction of new therapies targeting Bcl2 and the B cell receptor (BCR) signalling pathway that have revolutionised treatment for this disease^2^.

As a marker distinguishing between CLL cells that have undergone the germinal centre reaction, mutational status of the *IGHV* genes coding for the BCR is one of the strongest predictors of overall survival in this disease^1,3,4^. Importantly, it is found that BCRs on CLL cells from different patients can be virtually identical with respect to *IGHV* genes and sequences, indicating a potential common mechanism of disease pathogenesis in CLL involving a B cell populations with limited BCR heterogeneity and/or selection of the malignant clone by a limited set of antigenic determinants^5,6^. Indeed, mutational status of the *IGHV* genes confer antigen specificity; BCRs derived from unmutated genes are polyreactive whereas those derived from mutated genes are monoreactive^7^. Also, CLL cells with unmutated or mutated *IGHV* genes respond differently to BCR engagement^8^, a response thought governed by the ability of BCR to enter lipid raft structures^9^. Nevertheless, later studies linked BCR signalling capacity and evidence of engagement with markers of poor disease prognosis^10–13^. Interestingly, BCR signalling pathway proteins show high expression in CLL cells^14–17^, and some, including ZAP70, have been shown to have prognostic significance^18,19^. Considering that proteins such as Bruton’s tyrosine kinase (BTK) and Syk are also therapeutic targets^17,20,21^, it is possible that expression levels of other potential therapeutic targets within the BCR signalling pathway may also inform on CLL prognosis.

In this regard Lck may be an important consideration. Previous work performed by us^22^ and others^23,24^ show variable expression of this src-family kinase (SFK) in malignant cells from different patients with CLL without relation to disease parameters. Moreover, our work demonstrated Lck as a key mediator of BCR signalling in CLL cells, where expression levels of this SFK correspond with the strength of signal following BCR engagement^22^. Considering the demonstrated link between BCR signalling strength and poor disease outcome in CLL^10,13,25^, we reasoned that Lck levels may also correspond to disease outcome and warrant further investigation. Importantly, inhibition of Lck either using a specific inhibitor or siRNA-mediated knockdown blocks proximal and distal BCR signalling events in CLL cells, and removes their influence on overall cell survival. This phenomenon is reminiscent of the effects of 2 other BCR pathway inhibitors, idelalisib and ibrutinib, which inhibit PI3Kδ and Bruton’s tyrosine kinase (Btk), respectively^26^. The effectiveness of these agents in the therapy of CLL lies with their ability to promote lymphocytosis of malignant cells from proliferation centres^26^. Recent work from our lab^27^ suggests the mechanism used by idelalisib involves induction of lymphocyte egress through upregulation of the receptor for sphingosine 1-phosphate (S1PR1) and migration to sphingosine 1-phosphate (S1P), while others^28,29^ have demonstrated that the mechanism used by ibrutinib involves inhibition of chemokine- and BCR-induced integrin α4β1 adhesion to fibronectin and VCAM. Thus, Lck may be considered a biological target in the treatment of CLL in the same way targeting Btk and PI3Kδ are now.

The present study further defines Lck as a relevant target in CLL, and shows that inhibition of this SFK causes effects similar to those observed when CLL cells are treated with the phosphatidylinositol 3 kinase δ (PI3Kδ) inhibitor idelalisib. We also developed a robust flow cytometry-based technique to assess Lck expression, and used this assay to investigate the relationship between expression of this SFK and disease. However, our data show that Lck expression levels in CLL cells is unrelated to overall survival or time to first treatment. This result is surprising in light of our previous work showing Lck expression level is important for BCR signalling strength^22^, and implies that although Lck, like other kinases within the BCR signalling pathway, is targetable in CLL, measurement of its expression levels between patient samples is unlikely to be useful for stratifying BCR-targeted therapies.

## Results

### Lck expression in CLL cells varies between patient samples

**Figure 1:**
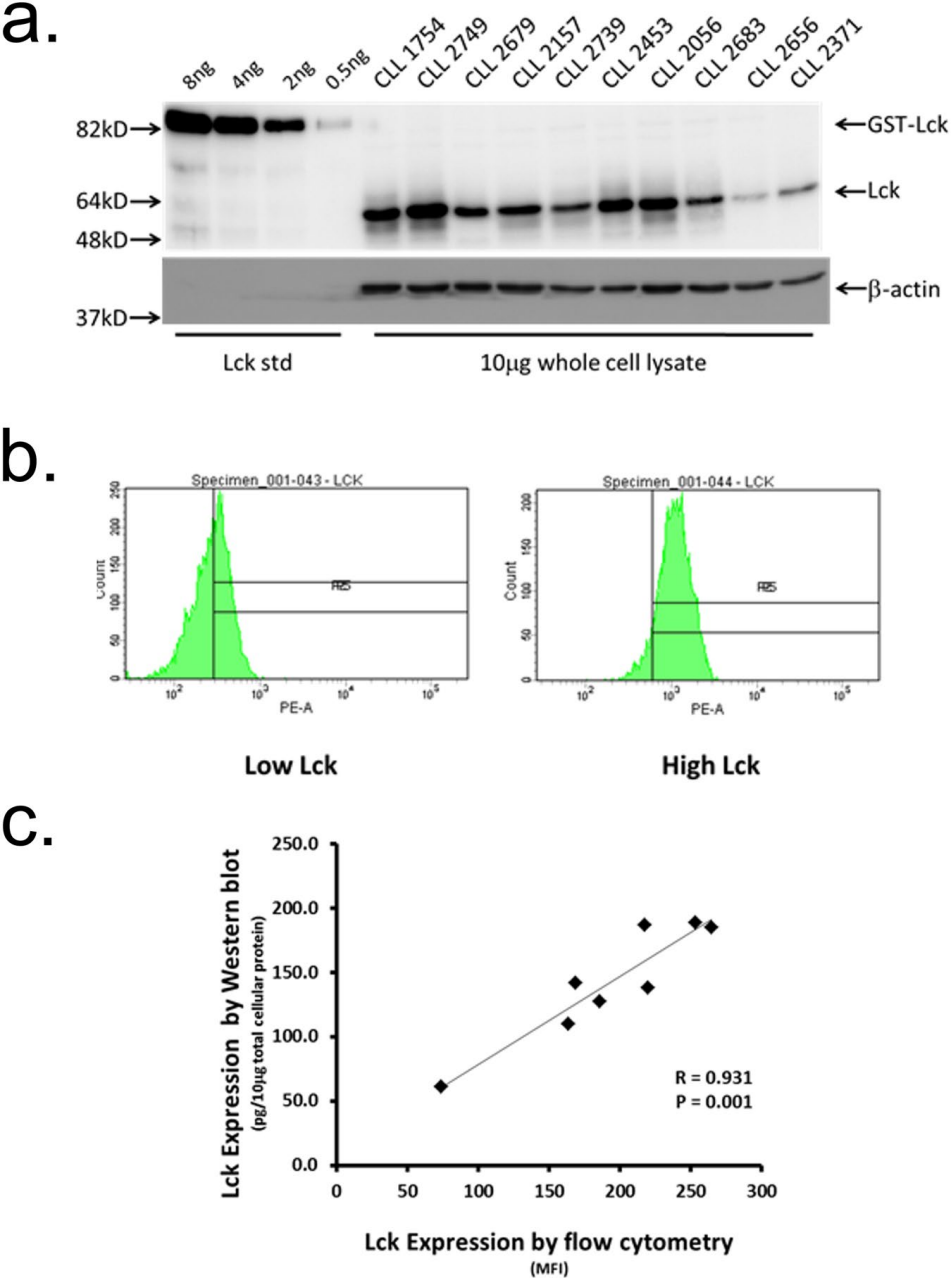
*Variable Lck expression levels in CLL cells determined by Western blot and flow cytometry*. (**a**) Western blot analysis of Lck expression in lysates of purified CLL cells. 10 μg of protein derived from lysates of CD19-purified CLL cells was separated by SDS-PAGE and immunoblots probed with either anti-Lck mAbs or anti-β-actin (as loading control). Varying amounts of purified recombinant Lck protein were used as standards to determine Lck levels in cell lysates. (**b**) Flow cytometry histograms of Lck expression in CLL cells derived from cases with known high and low levels. (**c**) Correlation of Lck expression in CLL cells determined by Western blot versus flow cytometry.

In our previous work^22^ we observed variance in Lck expression within isolated CLL cells from patient samples that corresponded to BCR signalling capacity. However, a key limitation of the approach used was that measurement of Lck expression levels could not be related to disease parameters over larger cohorts of patient samples. To increase the consistency of Lck measurement within the current study we developed a Western blot based method that quantitated expression levels of this SFK in purified CLL cells against standard amounts of recombinant Lck (Fig. 1a). We used this method to determine Lck expression in the malignant cells from 40 cases of CLL (Supplementary Table 1), and found a range of expression from very low (31.5 pg Lck/10 μg total cellular protein) to very high (235 pg Lck/10 μg total cellular protein) that was unrelated to *IGHV* mutation status.

Determination of Lck expression by Western blot is difficult and expensive owing to the need to purify CLL cells from T cell contamination, and then reliably quantitate Lck expression in these cells against recombinant Lck. To simplify this determination we developed a method to detect intracellular Lck by flow cytometry. Figure 1b and c show that flow cytometry can be used to detect different levels of Lck expression in CLL cells, and there is a high degree of correlation between the Western blot and flow cytometric methods for determining Lck expression. We therefore used flow cytometry to determine Lck expression in CLL cells from a second larger cohort of patient samples.

**Figure 2:**
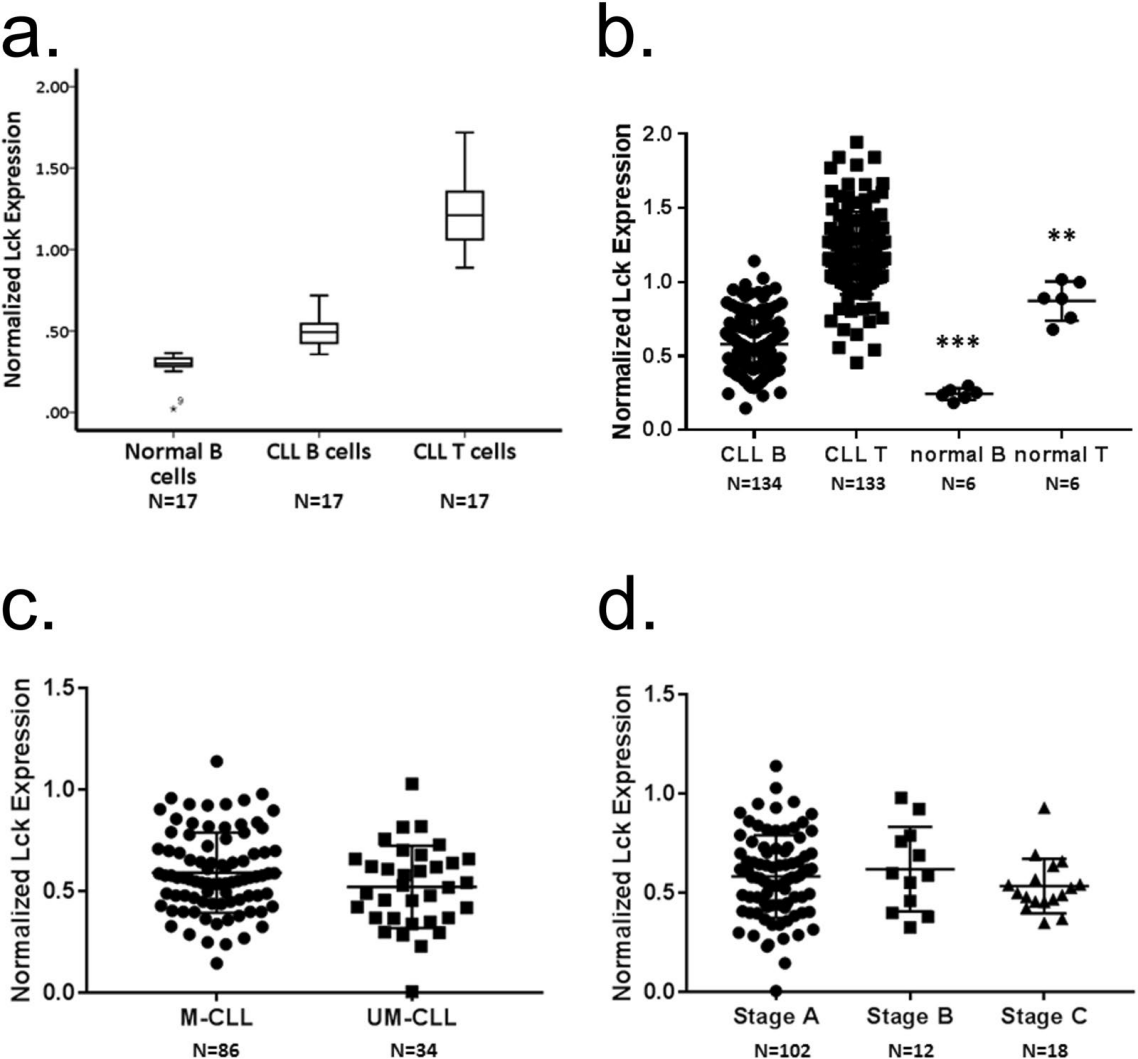
*Measurement of Lck expression in CLL cells of patient samples by flow cytometry*. (**a**) Box and whisker plots showing repeat measurements of Lck expression in normal B cells within a buffy coat samples from a single donor, and in T cells and malignant B cells from samples derived from a single patient with CLL. Lck expression in the cells illustrated by this experiment is normalised to that in normal T cells within the buffy coat sample. (**b**) Beeswarm plot of Lck expression in CLL cells, T cells from CLL patients, and B and T cells from normal patients. Statistical analysis was performed using a Mann-Whitney U test. “***” signifies a comparison of Lck expression between CLL cells and normal B cells, P < 0.001. “**” signifies a comparison of Lck expression between T cells from CLL patients and normal T cells, P = 0.008. (**c**) Comparison of Lck expression in UM- and M-CLL cells. (**d**) Comparison of Lck expression in CLL cells from patients with different stages of disease.

To control for experimental variability with respect to day-to-day measurement of Lck expression we used reference samples, a single CLL case and normal cells from a buffy coat, that were examined with each tranche of patient samples. Lck expression in cells was normalised for all analyses to that in normal T cells within the buffy coat reference sample. Figure 2a shows the results of 17 separate measurement experiments of Lck expression in the normal B cells of the buffy coat reference sample, and in CLL and T cells from the patient sample. Our method of approach showed a high degree of reproducibility, Lck measured at consistently low levels in normal B cells (0.31 ± 0.03). Since normal peripheral B cells do not express Lck^23^ we suggest that the value determined is a measure of background non-specific staining of the anti-Lck antibody we used. Measurement of Lck expression within CLL cells from the patient reference sample showed similar reproducibility (0.49 ± 0.10), and the value recorded was significantly higher (*P* < 0.001) than that observed for normal B cells indicating specific binding of the anti-Lck antibody. A surprising observation was Lck expression within the T cells of the CLL patient reference sample. The value observed here was reproducible (1.24 ± 0.23) and higher than that of normal T cells.

Analysis of Lck expression within CLL cells from the entire second cohort of 134 patient samples showed a broad range of values (Fig. 2b, Supplementary Table 2). Some cases of CLL expressed virtually undetectable levels of Lck (i.e. the values recorded were less than or equal to those associated with normal B cells), whereas Lck expression in others exceeded that in normal T cells. Generally, we observed that Lck expression within CLL cells from the patient cohort was significantly greater than that in normal B cells (P < 0.001). Further analysis of Lck expression in CLL cells from the patient cohort in relation to either *IGHV* mutation (Fig. 2c) or disease stage (Fig. 2d) showed no correlation, indicating that Lck levels cannot be used as a surrogate marker for the former and are likely not affected during the course of disease progression. Finally, in concordance with our finding using the single CLL case above we found that Lck expression in T cells from the CLL patient cohort was significantly higher than that observed in T cells from normal individuals (P = 0.008).

### Inhibition of Lck in CLL cells induces expression of S1PR1 and migration to S1P, but does not affect chemotaxis induced by CCL21

**Figure 3:**
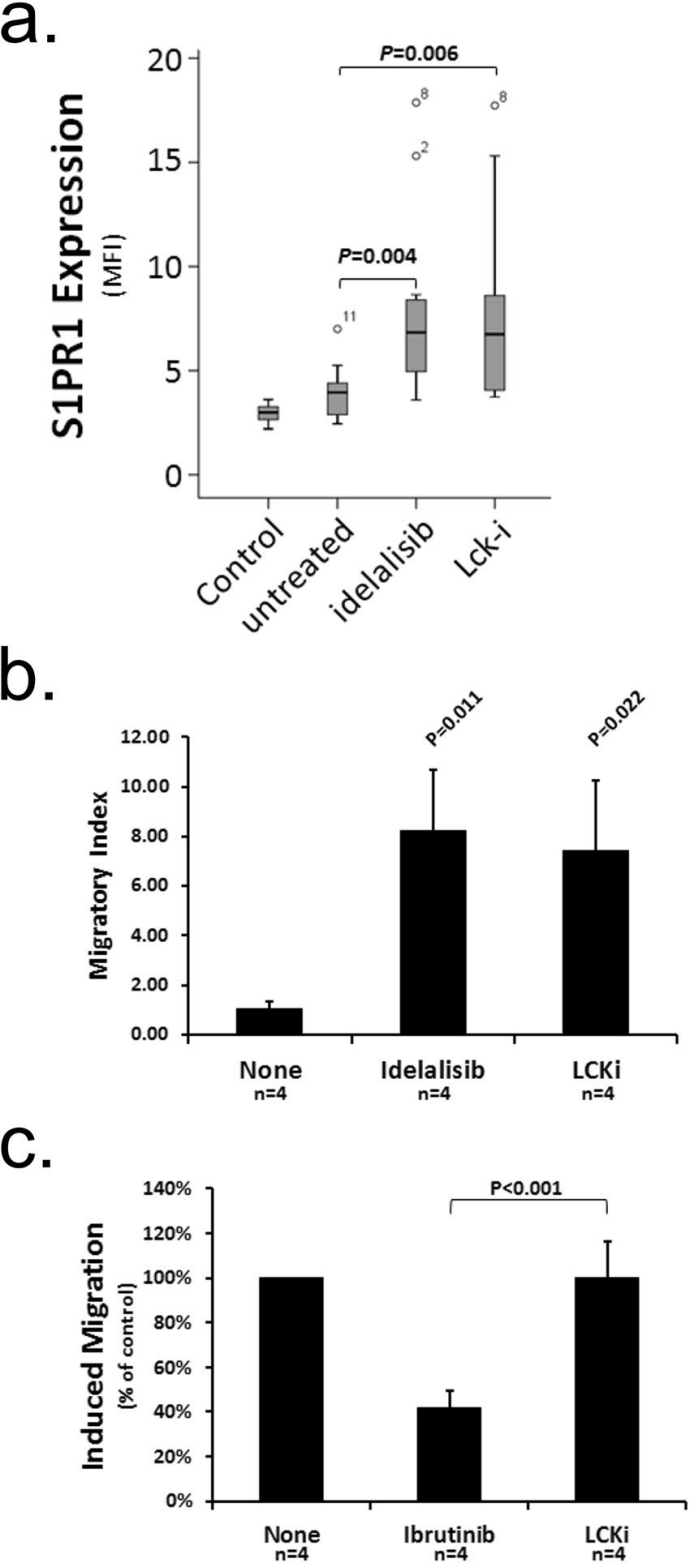
*Lck inhibition induces expression of S1PR1 on CLL cells and their migration toward S1P*. (**a**) CLL cells from 10 cases were examined immediately, or were cultured for 16 h with either idelalisib (1 μM), Lck-i (1 μM) or left untreated. All cells were examined by flow cytometry for S1PR1 levels. (**b**) CLL cells from four patients were incubated for 16 h with idelalisib (1 μM), Lck-i (1 μM) or left untreated (None), and then examined for migration toward S1P using HUVEC-coated transwells. The data are presented as migration index which is the quotient of the number of CD19 + cells transmigrating in the presence of S1P divided by that in its absence. (**c**) CLL cells from four patients were cultured with ibrutinib (1 μM), Lck-i (1 μM) or left untreated (None), and examined for migration toward CCL21 using HUVEC-coated transwells. Data are presented normalised to the level of CCL21-induced migration observed with untreated cells which is taken to be 100%. Statistical significance for all parts of this figure was determined using a Student’s t-test for paired data.

We next sought to determine whether Lck could be a relevant target in CLL similar to PI3Kδ due to the proximal role this SFK plays in the BCR signalling pathway. Figure 3a compares the effects of idelalisib and 4-amino-5-(4-phenoxyphenyl)-7H-pyrrolo[3,2d] pyrimidin -7-yl-cyclopentane (Lck-i) on S1PR1 expression on CLL cells. Both treatments induced significant upregulation of this receptor compared to untreated controls, the extent of which seeming unrelated to whether Lck was expressed at high or low levels within affected CLL cells (Supplementary Figure 2a). As expected, both treatments also induce significant enhancement of CLL migration to S1P (Fig. 3b), an effect that seemed reliant upon the level of S1PR1 expressed by CLL cells (Supplementary Figure 2b). As a control experiment, we compared the effects of Lck-i to those of ibrutinib in an assay measuring CCL21-induced transendothelial migration because our previous work^27^ showed the latter inhibits this process. This experiment showed CLL cell treatment with Lck-i was ineffective at inhibiting CCL21 migration, while treatment with ibrutinib significantly inhibited this process (Fig. 3c). This experiment indicates that kinases, like Lck, that are targeted by Lck-i do not function within signalling pathways controlling chemokine-induced migration of CLL cells in the same way kinases like Btk, targeted by ibrutinib, do. Within a broader interpretation the results of this section provide support for the notion of Lck as a relevant target because of the similar behaviour exhibited by CLL cells treated with Lck-i and idelalisib.

### Lck expression is associated with *in-vitro* and *in-vivo* sensitivity to chemotherapy

**Figure 4:**
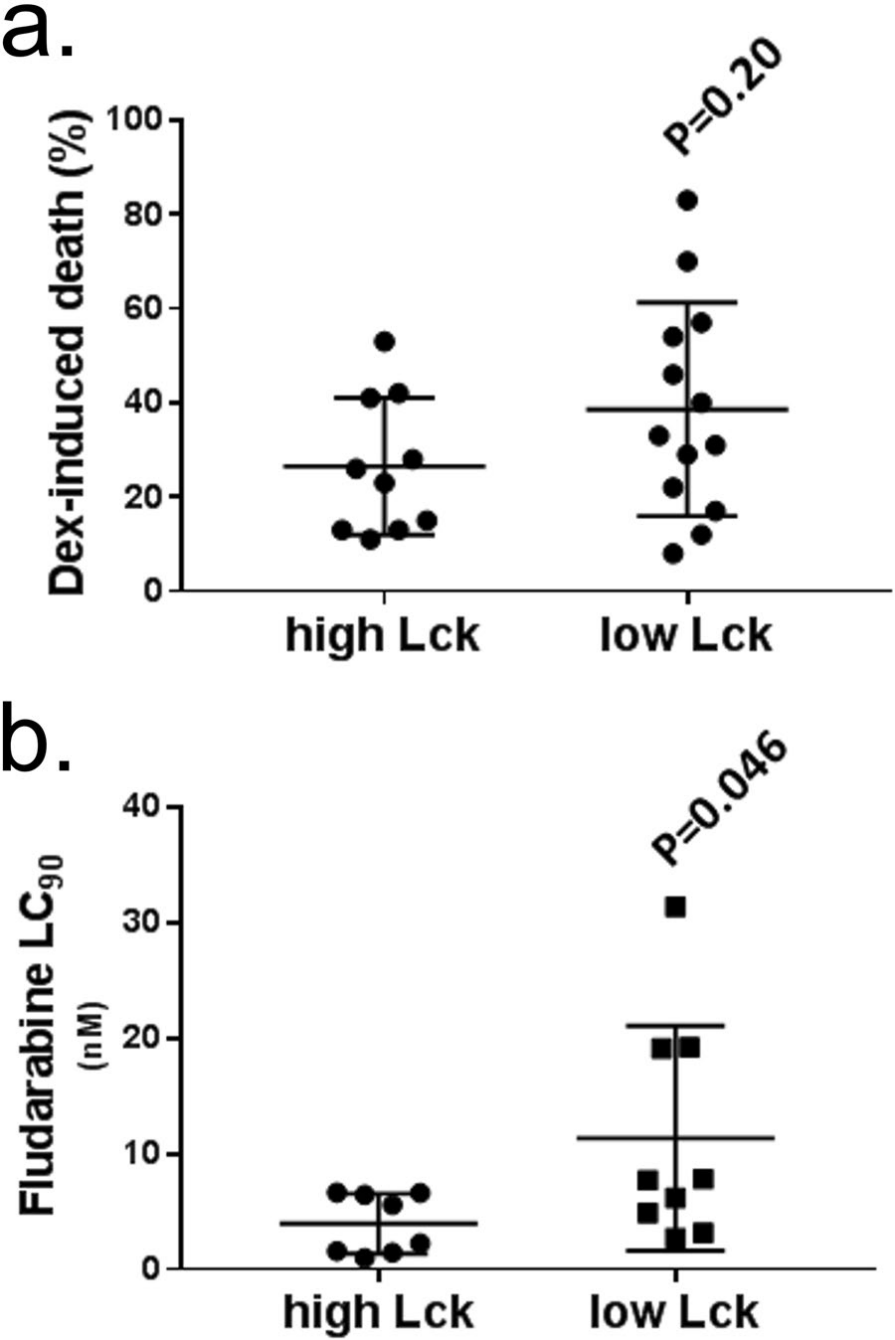
*Lck expression affects CLL cell sensitivity to fludarabine but not dexamethasone*. CLL cell sensitivity to (**a**) dexamethasone (presented as % induction of cell death) or (**b**) fludarabine (presented as concentration (nM) where 90% of cells have undergone apoptosis, LC_90_) was compared between CLL cells from cases in cohort 1 with high (>129 pg/10 μg total cellular protein) or low (<129 pg/10 μg total cellular protein) levels of Lck expression using data that corresponded to cases also used in Melarangi *et al*.^32^. Statistical significance for both parts of this figure was performed using a Mann-Whitney U-test.

Others have suggested that levels of Lck expression in haemic cells relate to responsiveness to chemotherapy^30,31^. To investigate this possibility we used previously generated data measuring malignant cell sensitivity to glucocorticoid and fludarabine treatment in CLL^32^, using only those cases for which we had data on Lck expression. Whereas CLL cell treatment with dexamethasone induced similar levels of cell death between Lck high and low expressing cases (Fig. 4a), we found that CLL cells expressing low levels of Lck required greater concentrations of fludarabine to achieve 90% cell death (LC_90_) than did those expressing high levels of Lck (Fig. 4b). This *in-vitro* resistance to fludarabine therapy is supported by *in-vivo* data showing that patients whose CLL cells expressed low levels of Lck were less likely to achieve a partial or complete response when being treated with this drug (Table 1). Thus, Lck expression in CLL cells may be an important indicator of disease response to therapy.

**Table 1:** Treatment response in relation to Lck expression within CLL cohort 2.

	NR	PR	CR	Total
Low Lck	7	20	4	31
High Lck	0	17	7	24

CLL cases divided into High and Low Lck expression, respectively defined as greater or lesser than the median value of normalized Lck expression (0.57), were analysed with respect to no response (NR), partial response (PR) and complete response (CR) to therapy consisting of chlorambucil and/or fludarabine and/or rituximab. Statistical analysis was performed using a χ^2^ test (P = 0.026, χ^2^ df = 7.289, 2).

### Lck expression is not related to disease prognosis

**Figure 5:**
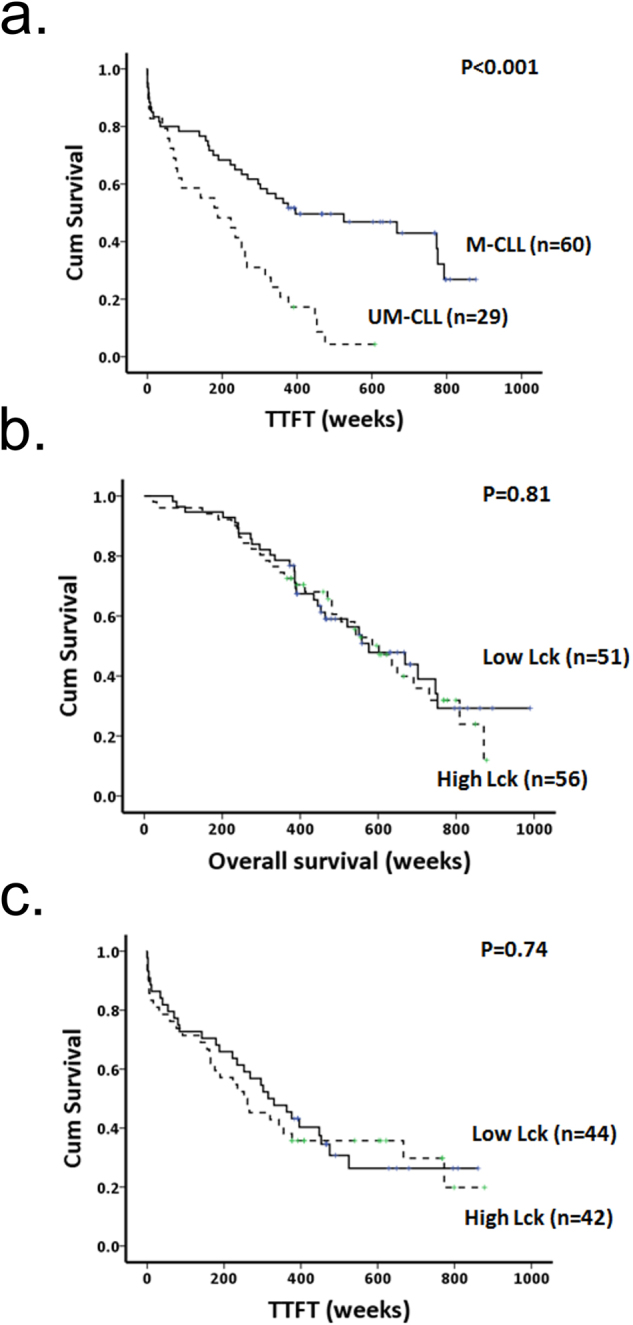
Lck expression in CLL cells is not predictive of overall survival or TTFT determined from disease diagnosis in a cohort of patient samples. (**a**) Kaplan Meier plots showing time to first treatment (TTFT) from disease diagnosis in patients having CLL cells with mutated (M) and unmutated (UM) *IGHV* genes. (**b** and **c**) Kaplan Meier plots showing overall survival and TTFT from disease diagnosis in patients having CLL cells with high or low levels of Lck expression. High and Low Lck expression is determined as being either greater or lesser than the median value (normalized Lck expression of 0.57) for all samples within the cohort.

**Figure 6:**
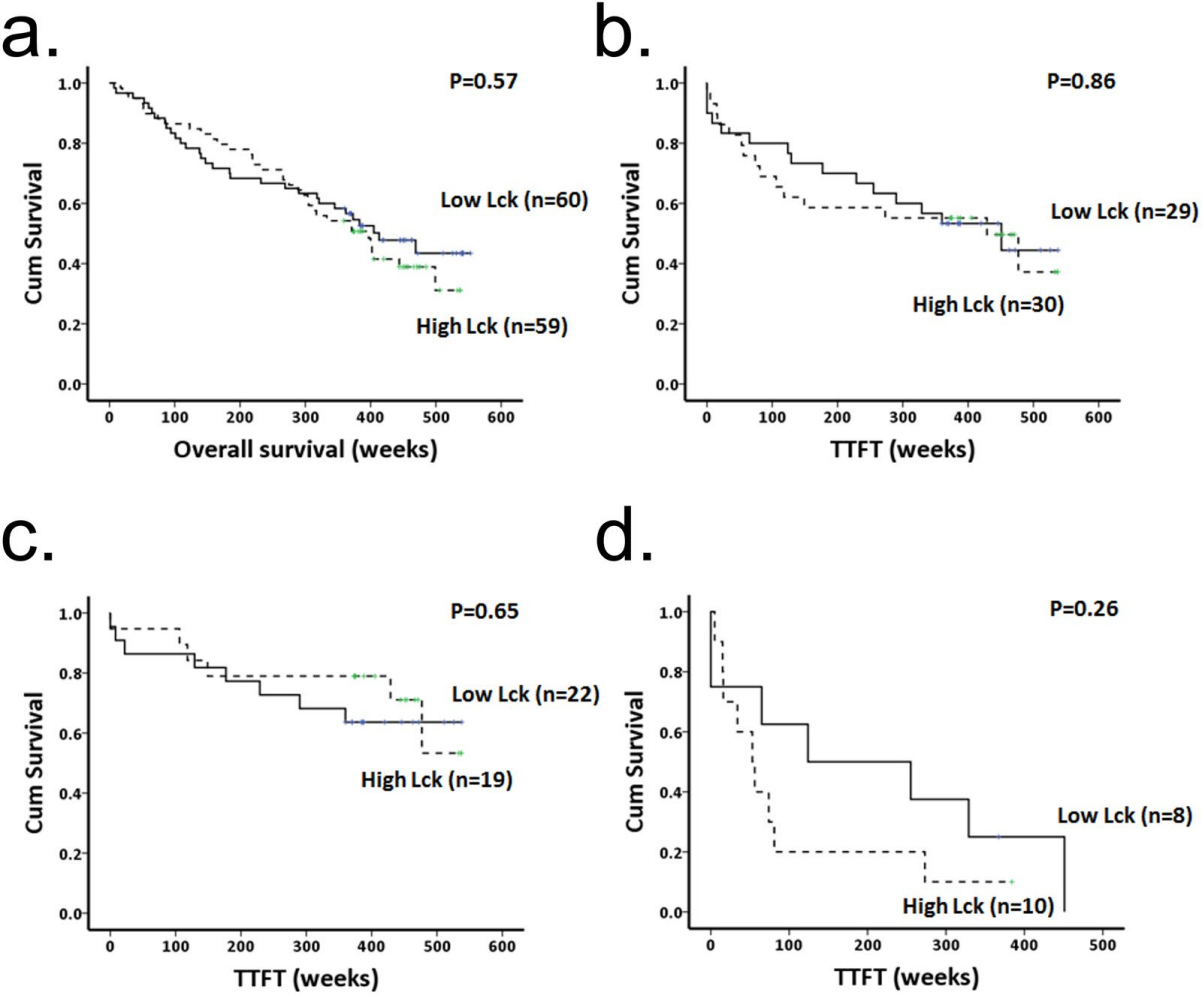
*Lck expression in CLL cells is not predictive of overall survival or TTFT determined from sampling*. (**a** and **b**) Kaplan Meier plots showing overall survival and TTFT from sampling in patients having CLL cells with high or low levels of Lck expression. (**c**) Kaplan Meier plot showing TTFT from sampling in patients having M-CLL cells with high or low levels of Lck expression. (**d**) Kaplan Meier plot showing TTFT from sampling in patients having UM-CLL cells with high or low levels of Lck expression. High and Low Lck expression is determined as being either greater or lesser than the median value (normalized Lck expression of 0.57) for all samples within the cohort.

The above data together with the demonstrated role of Lck as a mediator of BCR signalling capacity^22^ suggest that Lck expression within CLL cells may be useful as a predictor of prognosis and/or outcome for this disease. Figures 5 and 6 show our investigation of this hypothesis. In the first instance we validated our cohort of patient samples and show that *IGHV* mutation status is a predictor of time to first treatment (TTFT) (Fig. 5a). However, division of patient samples into CLL cases with high or low Lck expression did not relate either to overall survival or to TTFT (Fig. 5b and c). For this analysis we chose the cut off value of 0.57 to divide cases into high and low expressing cases because this corresponded approximately to the mean/median value of Lck expression in CLL cells regardless of whether we used the whole cohort of samples, or divided the cohort by M-/UM-CLL status (Fig. 2c) or by disease stage (Fig. 2d).

The data presented in Fig. 5 compare overall survival and TTFT from diagnosis. However, Lck expression potentially changes over the course of CLL, particularly if the cells are exposed to toll-like receptor antigens^33^. To take this into account we calculated overall survival and TTFT in patients using the sampling date rather than the date of diagnosis. Figure 6a and b show that no relationship exists between Lck expression and either of these two parameters within the cohort of patient samples we analysed. Furthermore, breakdown of the cohort into M- and UM-CLL cases and re-analysis of TTFT from sampling also failed to show significant relationship to high or low expression levels of Lck (Fig. 6c and d). Taken together, these data show that although Lck is a key regulator of BCR signalling^22^, expression levels of this kinase do not predict disease prognosis.

## Discussion

The aim of this study was to determine if Lck expression levels in CLL cells correlate with clinical outcome. This hypothesis is generated from previous work^22^ demonstrating that Lck is a key mediator of BCR signalling in CLL cells, and that BCR signalling strength in these cells is related to the level of this SFK that is present. However, we found no relationship between Lck expression and either overall survival or time to first treatment, even if patient samples were stratified into M- and UM-CLL subgroups.

The above finding is important relative to the work of others who have demonstrated a relationship between BCR signalling strength and progressive disease in CLL^10,13,25^. Given that several proteins within the BCR signalling pathway are reported expressed at higher levels in CLL than in normal B cells^14^, that some of these proteins such as Btk and Syk are also therapeutic targets and that this pathway plays an important role in the pathogenesis of this disease, it is not unreasonable to postulate a correlation between expression of pathway proteins, BCR signalling strength and disease outcome. Indeed, expression of ZAP70 is correlated with ability to signal through the BCR^34,35^, but its ability to enhance BCR signals is independent of its kinase activity^25,36^ indicating that inhibitors of ZAP70 may need to target protein interactions. High levels of Lyn expression are reported linked to shorter treatment-free survival in CLL^19^, and this kinase shows constitutive activation in CLL cells^16^. How this relates to BCR signalling strength is unclear because proximal signals such as phosphorylation of CD79 can be completely blocked by inhibitors of Lck which do not affect Lyn kinase activity^22^. Recently, a paper by Märklin *et al*.^37^ linked the anergic phenotype of CLL cells to NFAT2-driven expression of several genes including that coding for Lck. This paper is important because it suggests that high levels of Lck expression in CLL cells may predict favourable disease prognosis because BCR signalling anergy is associated with mutated *IGHV* genes^8^. The data provided by Märklin *et al*. support our findings that Lck is overexpressed in human CLL compared to normal B cells, however, whereas these same data show that low levels of Lck are associated with CLL cells from patients with aggressive disease, we find no such correlation of Lck expression with markers of poor prognosis in the cells from the cohorts of patient samples we analyzed. This discrepancy may be due to the nature of CLL cells studied by Märklin *et al*. where aggressive CLL refers to malignant cells derived from patients with Richter’s syndrome; we analysed no such cells in the current study. Moreover, the TCL mouse model of CLL used is similar to human UM-CLL with respect to the role of BCR on malignant cells in driving progressive disease^38–40^, and is an accepted paradigm of this type of disease^41^. In TCL1 CLL cells BCR signalling, although weak, is still present and conceivably mediated by Lck in the same way as we have demonstrated for human CLL cells^22^ because CD79 and Lck co-association can be demonstrated in BCR-stimulated cells^37^. With this in mind it is important to consider that *LCK* is not the only gene regulated by NFAT2 in the system studied^37^, and it is likely that the combination of regulated genes has functional consequence in producing BCR signalling anergy. In this context, our findings in the current study taken together with those of our previous work^22^ suggest that Lck expression is not *per se* responsible for mediating BCR anergy.

Interestingly, CLL cells expressing low levels of Lck required higher concentrations of fludarabine *in vitro* for effective induction of cell death than did CLL cells expressing high levels of Lck, and patients with these cells were less likely to have complete or partial response to this drug than patients with CLL cells expressing high levels of Lck. Thus, Lck expression may be an indicator of CLL cell resistance to chemotherapy and have importance for therapeutic decision making. Considering that fludarabine in combination with chlorambucil and rituximab is a front line therapy in CLL^42^, further study of this possibility is required to assess the value of Lck expression as a potential biomarker. Why Lck expression can be linked with disease resistance to chemotherapy but not have relationship to disease outcome in CLL is unclear. It is possible that Lck expression levels in CLL cells are affected by microenvironmental influences and may not be static in the same way other protein markers such as ZAP70 are. This notion is supported by studies showing that Toll-like receptor (TLR) agonists provide cultured CLL cells with increased resistance to fludarabine^43^ and induce downregulation of Lck expression^33^. Activation of Lck may also be important. We acknowledge that a weakness of our study is lack of data regarding the activation state of this SFK in the malignant cells of the patient cohorts we had available. pY^394^-Lck antibodies are now commercially available that were not when we began this study. Although this antibody may be a useful tool for the detection of active Lck, it should be noted that interpretation of results generated using this reagent may be problematic for the following reasons: (1). Lck shares significant homology with Blk within their respective peptide sequences containing the homologous pY^394^ residue. Therefore, in cells such as in CLL cells^44^ where both Blk and Lck are expressed, a positive result may not specifically indicate active Lck. (2). The need to calculate pY^394^-Lck/total Lck ratios specifically in CLL cells also presents problems in our study where we use flow cytometry to detect Lck expression. The pY^394^-Lck antibody available is sold unconjugated, making its use with other antibodies in flow cytometry technically difficult.

Our method using flow cytometry to assess Lck levels in CLL cells is robust. We tested this method against Western blot measurement of Lck expression to show there is a high degree of concordance between the two methods. Importantly, the Western blot technique we used was accurate and precise because Lck levels within CLL cell lysates were standardized against recombinant Lck, and because equal protein loading for each sample was ensured by protein assay and then confirmed by Western blotting for β-actin. A drawback of this approach is the requirement for CLL cells to be highly purified prior to lysis to eliminate the contribution of T and NK cell contamination to the level of Lck measured by Western blotting. Measuring Lck by flow cytometry is easier than by Western blot, particularly because CLL cells can be directly identified by antigen expression. An added advantage is that Lck can also be measured in accompanying T cells for comparison with known positives. Standardization in flow cytometry is applied by the inclusion of reference samples that are assessed with each lot of target CLL tests measured. In our case we included PBMCs isolated from a normal blood donor buffy coat, as well as cells from a CLL patient taken as a single sample. These reference samples were then analysed with each of the 17 lots of CLL cell tests from the cohort of patient samples we employed for this study. We normalised Lck expression in all cell types against that observed within the T cells of the reference PBMCs. As expected^45^, normal B cells within the reference PBMCs have very low levels of Lck. The mouse B-1 B cell subset is reported to express Lck, where it participates in regulating BCR signal transduction^46^. Although Lck expression in the human equivalent of B-1 cells is yet to be described, it is unlikely that our method detects any contribution because this subset of B cells comprises less than 10% of total circulating B cells^47^, and we did not specifically design our antibody panel to detect B-1 cells. Therefore, the low level of Lck expression we show for normal B cells can be taken as the lower limit of detection for our method. Interestingly, we observed that Lck levels in T cells from CLL patients was greater than that in T cells from normal individuals. This result is similar to observations made with ZAP70^48^, and is consistent with reports showing that this protein is expressed at higher levels in T cells from CLL patients than in T cells from normal donors^48,49^. Taken together, overexpression of Lck and ZAP70 in T cells of CLL patients could be reflective of either T cell dysfunction related to inability to form an immunological synapse that has been reported associated with this disease^50,51^, or to the development of T cell populations that are skewed towards a memory and/or senescent CD8^+^ T cell phenotype^52,53^.

A potential limitation of our study is the high proportion of M- to UM-CLL cases (approximately 2:1) within cohort 2 of the patient samples. Although this ratio is representative of disease in CLL^1^, it is possible that the high proportion of M-CLL cases may obscure the value of Lck expression with respect to disease prognosis. This is because Lck levels are likely not relevant to the potentially large proportion of cases within this subgroup where the malignant cells are reported to be anergic to BCR engagement^54^. Indeed, this notion finds resonance with observations that Lck levels have no prognostic value within the M-CLL subgroup. The probable influence of the large proportion of M-CLL cases is further supported by experiments comparing Lck expression between the M- and UM-CLL subgroups, and between CLL cells from patients with different stages of disease; no clear difference in Lck levels was observed. In addition, it is possible that high levels of Lck are associated with aggressive disease within the cohort of UM-CLL samples available to us (we observed clear poor prognosis associated with this subgroup), but any such interpretation must be taken with extreme caution until more cases can be added because our data are not significant in relation to Lck expression and clinical outcome within this subgroup. Our inability to find meaningful correlation could also be the result of choosing the Lck expression threshold close to the mean of the cohort. We do not think this is the case because reanalysis of the data to only consider very high and very low Lck expression did not show statistical significance with respect to overall survival or TTFT.

As a proximal regulator of BCR signalling it is plausible that inhibition of Lck will have clinical value, a concept that is enhanced by our previous demonstration that the Lck inhibitor we used has specificity for Lck but not for Lyn and that it blocks BCR signalling^22^. The experiments we present in this manuscript suggest that Lck inhibitors may be biologically useful because treatment of CLL cells with Lck-i promotes expression of S1PR1 to facilitate migration to S1P. The mechanism we propose for Lck-i is similar to that of idelalisib which we show in the current manuscript, and which we have previously shown^27^, induces upregulation of S1PR1 and migration to S1P in CLL cells. This notion is supported by observations reported here that treating CLL cells with Lck-i does not affect chemokine-induced migration, an effect that is similar to our reported observations of the effect of idelalisib on the same phenomenon^27^. In contrast, such migration is blocked by CLL cell treatment with ibrutinib, an effect that is reported to be mediated by inhibition of chemokine receptor-induced integrin α4β1 activation^27–29^, and a compound which we have shown neither influences expression of S1PR1 nor CLL cell migration to S1P^27^. Thus, Lck is not involved in chemokine receptor-induced signals, and Lck-i does not work in the same way as ibrutinib. The similarity of effects induced by CLL cell treatment with Lck-i and idelalisib does suggest that if the former was used *in vivo*, lymphocytosis as a result of the egress of CLL cells from proliferation centres would be observed.

In conclusion, although these studies cannot demonstrate relationship between Lck expression and clinical outcome in CLL, they are nevertheless important to our understanding of this disease. CLL cells are known to express various elements specific to the T cell receptor signalling pathway including Lck^22,23^, ZAP70^18,35^ and SLP76^55^. Interestingly, SLP76 is a scaffold protein and ZAP70 acts as a scaffold where it is expressed in CLL^34,36^, and both these proteins are substrates of Lck to suggest a potential relationship with respect to BCR signalling enhancement. In terms of the current study we were not able to test whether Lck expression levels refine disease prognosis in ZAP70 + CLL cases because too few cases were characterised for this protein. Considering that inhibition of Lck (with Lck-i) and of PI3Kδ (with idelalisib) produce mechanistically similar effects, determination of Lck expression in ZAP70 + CLL cases may be useful for patient stratification strategies to receive idelalisib.

## Materials and Methods

### Patient samples

Buffy coats used for this study were obtained from National Health Service Blood Transfusion. CLL cells were obtained from the peripheral blood taken from patients with informed consent and with the approval of the Liverpool and Hull Research Ethics Committees. Stored CLL cell samples from the Liverpool Leukaemia Biobank and Hull-York Medical School CLL Biobank were prepared using standard protocols as described previously^56,57^, and had a minimum viability of 80%. CLL sample usage and experiments performed on these samples were recorded in compliance with a Research Ethics Agreement overseen by the University of Liverpool and Royal Liverpool and Broadgreen NHS University Hospital Trust.

### Measurement of Lck expression by Western blot

Normal human B cells were purified from buffy coats using the B Cell Isolation kit II (MACS, Miltenyi Biotec, Surry, U.K.) according to the manufacturer’s instructions. Purity of the B cells isolated by negative selection was checked using flow cytometry and CD19 staining. In general, B cells treated in this way attained a purity that was greater than 90%. CLL cells were also negatively purified in a similar manner except that FITC-conjugated anti-CD3, -CD14, and -CD16 antibodies (BD Biosciences, Oxford, UK) were employed to separate T cells, NK cells, monocytes and macrophages using anti-FITC microbeads. CLL cell purity was checked using flow cytometry and CD5/CD19 staining. In general, CLL cells treated in this way attained a purity that was greater than 95%.

Cell pellets were prepared by lysing 1 × 10^7^ cells with 200 μl of lysis buffer (125 mM Tris pH6.8, 5 mM EDTA, 1%SDS). The lysates were sonicated to disrupt released DNA, incubated at 95 °C for 5–10 minutes and then centrifuged at 12,000 rpm for 5 minutes. The supernatant was transferred into a clean microtube and protein concentration was determined using the BioRad DC™ protein assay according to the manufacturer’s instructions (BioRad Laboratories Ltd, Hertfordshire U.K.). The lysates were stored at −20 °C until needed for further use.

To perform the Western blots 10 µg of total protein within cell lysates was loaded per lane of gel, and separated by sodium dodecyl sulphate-polyacrylamide gel electrophoresis using 10% polyacrylamide gels. The separated proteins were then electroblotted to Immobilon^TM^ membranes (Millipore, Fisher Scientific UK Ltd, Loughborough, UK). The membranes where then incubated for 1 h with blocking buffer [2.5% ECL advance blocking reagent (Millipore, Fisher Scientific UK Ltd, Loughborough, UK) in TBS-T (150 mM NaCl, 25 mM Tris pH 7.5, 0.1% Tween 20)], and then probed overnight at 4 °C with anti-Lck mAbs (clone 3A5, Santa Cruz Biotechnology, Insight Biotechnology Ltd, Wembley U.K.) diluted 1:1000 with blocking buffer. Following a second 1 hour incubation with horse radish peroxidase (HRP)-conjugated anti-mouse antibodies (diluted 1/5000 in blocking buffer), the membranes were thoroughly washed and then developed with ECL-advance Western Blotting Detection reagents (Millipore, Fisher Scientific UK Ltd, Loughborough, UK). Chemiluminescence was read using a Fujifilm LAS-1000 and Lck within cell lysates was quantitated against a standard curve of known amounts of recombinant Lck (R&D Systems Abingdon U.K.).

### Measurement of Lck expression by flow cytometry

5 × 10^6^ cells were fixed and permeabilised using Phosflow™ fix and permeabilisation buffer II (BD) according to the manufacturer’s instructions. Cells were then incubated with incubated with PE-conjugated anti-Lck (clone MOL 171), FITC-conjugated anti-CD3, APC-conjugated anti-CD5 and PerCPCy5.5-conjugated CD19 mAbs (all from Becton Dickinson, Abbingdon, UK). The stained cells were then washed with modified PBS and analysed using a BD Fortessa flow cytometer. Lck expression in target cells (measured as mean fluorescence) was standardised against that in normal T cells within a sample from a single donor. This sample, derived from a buffy coat, was used, along with samples from a single CLL case, as a staining control to insure experimental reproducibility.

### Measurement of S1PR1 expression by flow cytometry

Cells were simultaneously stained with directly conjugated mAbs to S1PR1 (clone 218713, PE conjugated; R&D Systems) and CD19 (PerCP-Cy5.5 conjugated; BD Biosciences, Oxford, U.K.) together with appropriate isotypic control Abs and analysed by multicolor flow cytometry. The percentage and mean fluorescence intensity for S1PR1 was determined on CD19+ cells. In addition, viability of the cells after culture was assessed using propidium iodide. None of the drugs used decreased the viability of CLL cells following culture. Because cells were cultured in conditions to minimize cell death, viability was >75% in the majority of cases.

### Migration assays

HUVECs were grown to confluence on the inserts of Transwell plates (5 μm pore size; Corning, High Wycombe, U.K.). Chemokines, S1P (Sigma-Aldrich) or CCL21 (R&D Systems), were added to the bottom wells at concentrations (100 ng/ml and 1 μg/ml, respectively) shown to induce maximum migration^27^. CLL cells were added to the inserts, and the number of B cells that had migrated to the bottom wells was counted after 6 h incubation. The migration index (number of CD19+ cells transmigrating with chemokine divided by number of cells transmigrating in the absence of chemokine) was then calculated. Idelalisib, ibrutinib and 4-amino-5-(4-phenoxyphenyl)-7H-pyrrolo[3,2d] pyrimidin -7-yl-cyclopentane (Lck-i) were all used at a final concentration of 1 μM.

### Statistical analysis

Statistical analyses for this study were performed using SPSSv12™ and Microsoft Excel™ software packages.

### Data availability

All data generated or analysed during this study are included in this published article (and its Supplementary Information files).

## Electronic supplementary material

Supplementary Figures

Dataset 1

Dataset 2
