# Supplementary material for: Lck is a relevant target in chronic lymphocytic leukaemia cells whose expression variance is unrelated to disease outcome
Source: Sci Rep. 2017 Dec 1;7:16784. doi: 10.1038/s41598-017-17021-w (PMC5711840; doi:10.1038/s41598-017-17021-w)
Supplement: Supplementary file 1 — Supplementary Figures [file 41598_2017_17021_MOESM1_ESM.pdf]

Supplementary Figures

<u>Lck is a relevant target in chronic lymphocytic leukaemia cells whose expression variance is unrelated to disease outcome</u>

Kathleen J Till, John C Allen, Fatima Talab, Ke Lin, David Allsup, Lynn Cawkwell, Alison Bentley, Ingo Ringshausen, Andrew D. Duckworth, Andrew R. Pettitt, Nagesh Kalakonda, Joseph R Slupsky

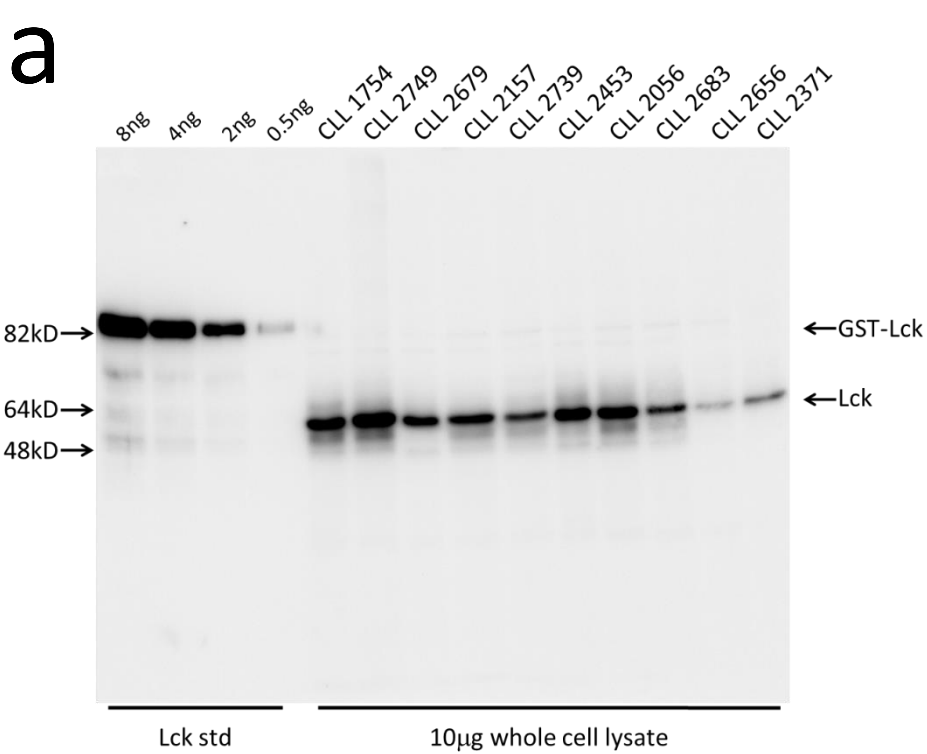

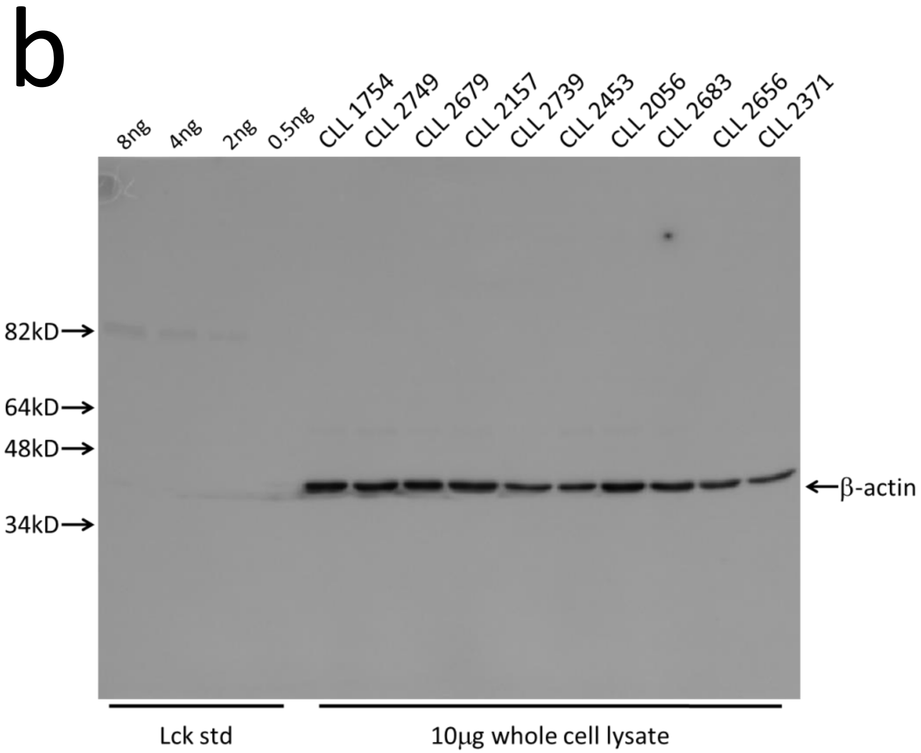

Supplementary Figure 1. Uncropped Western blots for **a**.) Lck and for **b**.) β-actin represented in manuscript Figure 1a.

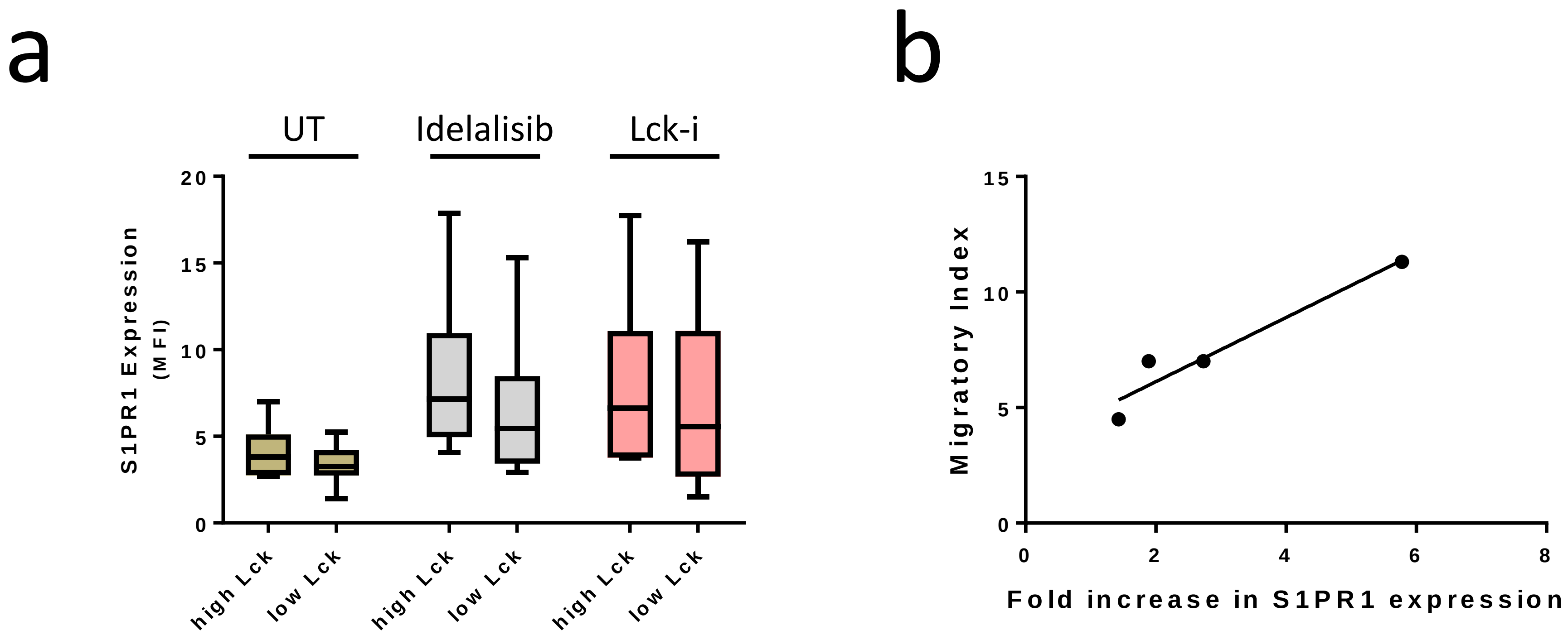

Supplementary Figure 2. Relationship between Lck and S1PR1 expression. a.) S1PR1 expression on CLL cells, cultured for 16h with either idelalisib (1 μM), Lck-i (1μM) or left untreated , was determined by flow cytometry and compared between cells expressing high (n=6) and low (n=10) levels of Lck. b.) Correlation between CLL cell migration (presented as migration index) to S1P and fold increase in S1PR1 expression in cells treated with Lck-i (1 μM) for 16h.
